# Association of Emotional Labor and Occupational Stressors with Depressive Symptoms among Women Sales Workers at a Clothing Shopping Mall in the Republic of Korea: A Cross-Sectional Study

**DOI:** 10.3390/ijerph14121440

**Published:** 2017-11-23

**Authors:** Yuh-Jin Chung, Woo-Chul Jung, Hyunjoo Kim, Seong-Sik Cho

**Affiliations:** 1 Department of Psychiatry, Mentors Hospital, Seoul 02617, Korea; redbaba@hanmail.net; 2 Department of Occupational and Environmental Medicine, Korea University Ansan Hospital, Ansan 15459, Korea; jwcdk@hanmail.net; 3 Department of Occupational and Environmental Medicine, Ewha Womans University Mokdong Hospital, Seoul 07985, Korea; 4 Department of Occupational and Environmental Medicine, Hallym University Sacred Heart Hospital, Anyang 14068, Korea; 0361pt@hanmail.net

**Keywords:** emotional labor, job insecurity, social support, workplace mistreatment depressive symptoms, women sales workers

## Abstract

In the distribution service industry, sales people often experience multiple occupational stressors such as excessive emotional labor, workplace mistreatment, and job insecurity. The present study aimed to explore the associations of these stressors with depressive symptoms among women sales workers at a clothing shopping mall in Korea. A cross sectional study was conducted on 583 women who consist of clothing sales workers and manual workers using a structured questionnaire to assess demographic factors, occupational stressors, and depressive symptoms. Multiple regression analyses were performed to explore the association of these stressors with depressive symptoms. Scores for job stress subscales such as job demand, job control, and job insecurity were higher among sales workers than among manual workers (*p* < 0.01). The multiple regression analysis revealed the association between occupation and depressive symptoms after controlling for age, educational level, cohabiting status, and occupational stressors (sβ = 0.08, *p* = 0.04). A significant interaction effect between occupation and social support was also observed in this model (sβ = −0.09, *p* = 0.02). The multiple regression analysis stratified by occupation showed that job demand, job insecurity, and workplace mistreatment were significantly associated with depressive symptoms in both occupations (*p* < 0.05), although the strength of statistical associations were slightly different. We found negative associations of social support (sβ = −0.22, *p* < 0.01) and emotional effort (sβ = −0.17, *p* < 0.01) with depressive symptoms in another multiple regression model for sales workers. Emotional dissonance (sβ = 0.23, *p* < 0.01) showed positive association with depressive symptoms in this model. The result of this study indicated that reducing occupational stressors would be effective for women sales workers to prevent depressive symptoms. In particular, promoting social support could be the most effective way to promote women sales workers’ mental health.

## 1. Introduction

Emotional labor is a growing issue in workplace mental health. According to Hochschild [1], emotional labor was defined as “the management of feeling to create a publicly observable facial and bodily display” in service work. In other words, it referred to how workers must manage their emotions to appeal to customers. De Castro and colleagues suggested three principal characteristics of emotional labor [2]; first, it requires face-to-face or voice-to-voice contact with customers, and thus it often took place in settings such as small shops, shopping malls, banks, hotels, and restaurants. Second, it requires workers produce an emotional state necessary for their specific jobs and could be an essential component of their job duties. Finally, employers often train or supervise employees to control their emotional activities. 

Given that activities in the service economy are proliferating extensively, the number of workers whose tasks mainly involve emotional labor is increasing globally as well as in South Korea. Indeed, the proportion of workers involved in interactive service work for over 50% of their working hours was estimated to be around 41.8% according to Kim and colleagues [3].

There has been a considerable amount of literature focusing on the negative effects of emotional labor, which include depression [4], somatoform symptoms [5], anxiety disorders [6,7], and burn out [8,9]. Among these negative health outcomes, depressive symptoms are especially detrimental for the workplace. Specifically, depression could negatively influence job performance, which could in turn lead to functional impairment [10]. Furthermore, depression at the workplace could result in reduced work productivity and socioeconomic losses to companies or individual workers [11]. 

A national UK survey reported that there are occupational differences in common mental disorders and women sales occupations showed the highest prevalence of mental disorder (20%) compared to the 13% overall prevalence in all adults. The study suggested that once the causes are better understood, then effective interventions can be put in place to improve the psychosocial and physical conditions at work although the reasons for these differences are complex [12]. According to a recent systematic review article, a number of well-known factors could lead to depressive symptoms in the workplace. Moderately strong evidence was found for job strain, low decision latitude, and bullying having significant impact on the development of depressive symptoms. Limited evidence was shown for psychological demands, effort reward imbalance, low support, unfavorable social climate, lack of work justice, conflicts, limited skill discretion, job insecurity, and long working hours [13]. Moreover, service workers were often exposed not only to excessive emotional labor, but also workplace mistreatment by customers—such as incivility [14] and sexual harassment [15]—which had both been found to be risk factors of depressive symptoms. Because workers in these industries have direct contact with customers on a regular basis, they often must interact with angry, hostile, or uncooperative customers. These situation can lead workers to emotional exhaustion and depressive symptoms.

To summarize the above discussion, this study aimed to explore the predictors of depressive symptom among sales workers, and to provide evidences for establishing strategies for their mental health. The main objectives of this study are as follows: (1) to examine the association of occupation with depressive symptoms, i.e., whether sales workers experience more depressive symptoms compared to manual workers; (2) to examine differences and similarities in the association of occupational stressors with depressive symptoms among sales workers and manual workers; and (3) to examine the association of emotional labor with depressive symptoms among sales workers. 

## 2. Methods

### 2.1. Study Design and Ethics

A cross sectional study was conducted in in November 2012. All participants gave their informed consent. The study procedure was approved by the ethics committee of the Faculty of Medicine of Dankook University Hospital (Approval No. 2012-10-010). 

### 2.2. Participants

The study population was women workers aged 30–49 years comprised of 350 sales workers from 243 shops in a clothing shopping mall and 233 manual workers from 102 small-scale manufacturing companies. Manual workers were used as a control group because they were not exposed to emotional labor that stems from interfacing with customers. All the study population was sampled from participants in 2012 Seoul Women’s Health Promotion Project. In the project, the investigator received permission from the employers in advance and visited the workplace. Interviews were conducted at rest time to voluntary workers who work in the field at the time of the visit [16].

### 2.3. Measurement

The questionnaire consisted of demographic factors; occupational stressors such as workplace mistreatment, emotional labor, and job stress; and depressive symptoms. Demographic factors were identified through the following questions; “When is your birth year?”, “What kind of school did you graduate from?”, and “Do you live with someone else in your current residence?”. Workplace mistreatment was measured using single question: “Have you ever experienced verbal violence, unwanted sexual attention, and humiliating or threatening behavior in your workplace for the past one month?”. Emotional labor was measured by the questionnaire of Chu’s version [17] based on Morris and Feldman’s theory [18]. We used the questionnaire because Morris and Feldman suggested ‘emotional dissonance’ as a component of emotional labor, which had been described mainly by the frequency of emotional expression until then.

The result of factor analysis demonstrates two subscales of emotional effort and dissonance. Emotional effort means how much effort must be put into expressing the feelings required by the company to deal with customers, and was measured by three following statements: (1) I have to hide my true feelings for my job; (2) I make an effort to be kind for the customers or clients; (3) I make an effort not to show negative emotions. Emotional dissonance was the difference between workers’ own feelings and the emotions workers have to express, and was measured with the five following statements: (4) I have to pretend to smile; (5) I am stressed with not expressing true feelings; (6) Smiling at every situation is very difficult task; (7) There are differences between true feelings and emotional expressions; and (8) I am confused at the difference between true feelings and required emotional expressions. The above items were answered by four-point scale according to their agreement levels ranged 0 to 3. The short form of the Korean Occupational Stress Scale [19] was used to evaluate four dimensions of job stress; job demand, job control, job insecurity, and lack of social support. Each item was rated using a four-point scale. The item scores of emotional labor and Korean Occupational Stress Scale were summed for each subscale. The higher summation scores mean the more exposure to the occupational stressors. The Korean version of the Center for Epidemiologic Studies Depression Scale (CES-D) [20], which comprises 20 items answered by four-point scale from 0 to 3 points according to their agreement levels, was used to measure depressive symptoms. The summation scores on the CES-D range from 0 to 60, in which higher scores suggest a greater presence of depressive symptoms.

### 2.4. Statistical Analysis

SPSS 22.0 (IBM, New York, NY, USA) was used for statistical analyses. We presented descriptive statistics for depressive symptoms and various explanatory variables. Independent *t*-test and chi square tests were done to identify the differences of these variables between sales and manual workers. Pearson’s correlation coefficients were calculated for the variables. A *p*-value < 0.05 was considered to be statistically significant. Hierarchical regression analysis was utilized in order to examine whether the occupation had an association with depressive symptom. A multiple regression model stratified by occupation was constructed to identify the associations of occupational stressors and emotional labor with depressive symptoms in each occupation. Another multiple regression analysis was done to reveal the associations of emotional effort and emotional dissonance with depressive symptoms among sales workers.

## 3. Results

Table 1 lists the descriptive statistics, the Cronbach’s α values, and correlations among the study variables. More women sales workers had a high educational level and lived alone compared to manual workers. Scores for depressive symptoms, job demand, job control, and job insecurity were significantly higher among sales workers than among manual workers. These differences were statistically significant (*p* < 0.05), whereas there were no differences in age and social support. 11.1% of sales workers had experienced workplace mistreatment for past one month, compared to 6.1% of manual workers (*p* < 0.05). Significant positive correlations were observed between depressive symptoms and occupational stressors such as job demand, job insecurity, and workplace mistreatment in both occupations (Table 1). 

Table 2 shows the result of multiple regression analysis. Occupation with age, cohabiting status, and educational level were entered in Step 1 of each regression. Occupational stressors such as workplace mistreatment, job demand, job control, lack of social support, and job instability were entered simultaneously in Step 2, with the interaction term occupation × job introduced in Step 3. Before conducting the regressions, residual plots were used to test normality, linearity and equality of variances, with no violations being found. Interaction terms were computed as the product of the centered scores. The multiple regression analysis revealed the association between occupation and depressive symptoms after controlling for age, educational level, cohabiting status, and occupational stressors (sβ = 0.08, *p* < 0.05). A significant interaction effect between occupation and social support was also observed in this model (sβ = −0.09, *p* < 0.05) (Table 2).

The multiple regression analysis stratified by occupation showed that job demand (sβ = 0.12, *p* < 0.05), job insecurity (sβ = 0.33, *p* < 0.01) and workplace mistreatment (sβ = 0.24, *p* < 0.01) were significantly associated with depressive symptoms in sales workers’ model. The manual workers’ model also showed the associations of these occupational stressors with depressive symptoms, although the strength of statistical associations were slightly different. We found negative associations of social support (sβ = −0.22, *p* < 0.01) and emotional effort (sβ = −0.17, *p* < 0.01 with depressive symptoms in another multiple regression model for sales workers. Emotional dissonance (sβ = 0.23, *p* < 0.01) showed positive association with depressive symptoms in this model (Table 3).

## 4. Discussion

This study showed that sales workers had more depressive symptoms than manual workers after controlling for various confounders. However, caution must be exercised in comparing the prevalence of depressive symptoms in this study with that of previous studies. Depression has a number of categorizations, such as major depressive disorder, minor depressive disorder, subsyndromal depression, and depressive symptoms. Among them, depressive symptoms were more prevalent compared to the other categories of depression [21].

This study result was consistent with a large cross-sectional study on 8522 workers by Cho and colleagues. Emotional labor was reported as an occupational risk factor for depressive symptoms because people involved in industries with more exposure to emotional labor—such as recreational, cultural, and sporting activities; hotels and restaurants; and wholesale and retail—had significantly greater risks of depressive symptoms compared to those involved in industries offering “business activities” [22].

It is interesting that occupation and social support showed interaction effect for predicting depressive symptoms. Sales workers with social support, which is an important protective factor against depressive symptoms, showed a negative association with depressive symptoms in this study. The role of social support in preventing depressive symptoms is well-known. Paterniti and colleagues [23] found that low social support at work had been predictive of increased CES-D scores. In another study, inadequate social support was an important risk factor for depressive symptoms [22]. However, it is difficult for sales workers to obtain social support in their workplace because they tend to work for long hours alone or with one coworker. Thus, reducing work hours is recommended so that sales workers can establish individual support systems outside of the workplace. This could also contribute to workers’ recovery from occupational stress.

This study found that the occupational stressors associated with depressive symptoms in both occupations were workplace mistreatment, job demand, and job insecurity. However, the magnitude of their association with depressive symptoms varied slightly according to the occupation. In sales workers, the most important factor based on the effect size was job insecurity, followed by social support, workplace mistreatment, and job demand. Social support showed a significant negative association with depressive symptom only among sales workers. It is well-known that job insecurity due to nonstandard work has a harmful effect on workers’ physical and mental health [24,25]. In one study on 3851 workers, there was an higher probability of producing depressive symptoms among those who agreed that their job security was poor, even after adjusting for various confounders [26]. Furthermore, according to Kim and colleagues [27], nonstandard work status was associated with poor mental health after adjusting for socioeconomic status and health behaviors. As the number of nonstandard workers is growing globally, job insecurity has been an increasingly prevalent issue since the 1997 financial crisis in South Korea.

This study found that workplace mistreatment was another noticeable factor related to depressive symptoms among women sales workers. This finding is consistent with the report of Langlois [28] that workplace mistreatment was associated with depressive symptoms as well as burnout. Although there are few studies on the workplace mistreatment, we believe it to be a workplace hazard widely exposed among sales workers. In one survey, call center employees reported an average of seven hostile calls per day [14]. Although some studies have examined the relationship between customer incivility and employees’ general well-being [29,30], few studies have detailed the association between workplace mistreatment and depressive symptoms.

It is well known that job demand was related to depressive symptom in many occupations as mentioned earlier. However, job demand showed relatively weak association with depressive symptoms among sales workers than among manual workers. Furthermore, the association became statistically not significant, when emotional effort and emotional dissonance were added in the multiple regression model for sales workers. It suggests that there may be other occupational stressors that play an important role in the development of depressive symptoms among sales workers.

One of the remarkable findings of this study was that emotional dissonance had a positive association with depressive symptoms among sales workers, whereas emotional effort showed a negative association with depressive symptoms in this population. Morris and Feldman [18] noted that emotional dissonance was a positive predictor of emotional exhaustion and a negative predictor of job satisfaction. There were considerable evidences that emotional dissonance at least partly mediates the negative consequences of emotional labor including depression [31]. Emotional effort itself may not a risk factor for depressive symptom, but rather it can be relieved through efforts to express positive emotions required during sales work. On the other hand, it may be difficult to make emotional efforts if depressed. Further research with longitudinal design is needed to explore the meaning of this finding from this cross sectional study.

From the above findings, the higher scores of depressive symptoms among sales workers than among manual workers could be explained by exposure to multiple occupational stressors such emotional dissonance, workplace mistreatment, job insecurity, and lack of social support. Although not presented as a result of the study, no interaction between these occupational stressors was observed in our study. However, some authors have insisted that emotional labor interacts with other occupational stressors [32]. Future studies are needed to explore this possibility of the interaction.

There were several limitations in this study. First, there could be selection bias since this study used a convenient sample. However, this result might be generalized to worksites with similar characteristics because these sample were from a lot of workplace over 100 sites and the sample size was relatively large compared to previous studies. If not so, it is worth reporting considering it is very difficult to approach on these women workers scattered in sales shops and small scale manufacturing companies. Second, causal relationships cannot be established since it was a cross-sectional study. In other words, it is possible that depressive symptoms influence the perception of occupational stressors. Finally, the study variables were measured by self-report. Depending on their personality, workers can perceive different degrees of burden in the same position. Moreover, we could not measure depressive symptoms using clinician’s observations and interviews. However, few related studies have been able to diagnose depressive disorders by professionals. 

Despite these limitations, this study has considerable significance in that it showed the higher score of depressive symptoms among the sales workers who performed emotional labor compared with the manual workers as the control group. The results of this study are worth reporting considering it is very difficult to approach on these women workers scattered in sales shops and small scale manufacturing companies. In addition, similarities and differences in the factors related to depressive symptoms among sales workers and manual workers were identified. Finally, we found that emotional dissonance was one of the important occupational stressors related to depressive symptoms in sales workers. These results could provide a basis for the establishment of strategies for promoting mental health of women sales workers.

## 5. Conclusions

Sales workers had more depressive symptoms than manual workers. Sales workers were exposed to multiple occupational stressors which were risk factors of depressive symptoms. The significant predictors of depressive symptoms were emotional dissonance, workplace mistreatment, and job insecurity among sales workers. Social support showed a negative association with depressive symptoms especially among sales workers. The results of this study suggest that reducing exposure to these occupational stressors would be an effective strategy for preventing depressive symptoms among sales workers. In particular, promoting social support could be the most effective way to promote women sales workers’ mental health.

## Figures and Tables

**Table 1:** Means, standard deviations, correlations, and Cronbach’s α for variables.

	General Characteristics	Sales Workers (*n* = 350)	Manual Workers (*n* = 233)	1	2	3	4	5	6	7	8	9	10	11
1	Age (years, M (SD))	41.1 (5.5)	41.8 (5.4)	-	−0.31 **	0.47 **	−0.08	−0.21 **	−0.21 **	−0.10 **	−0.17 **	−0.02		
2	Education > high school (n (%)) ^†^	88 (25.1)	13 (14.2)	−0.14 *	-	−0.23 **	0.06	0.15 *	0.15 *	0.04	0.01	0.01		
3	Living alone (n (%)) ^†^	72 (20.6)	32 (13.7)	0.39 **	−0.06 **	-	−0.20 **	−0.20 **	−0.13	0.02	−0.10	−0.06		
4	Depressive symptoms (M (SD)) ^‡^	18.8 (8.4)	15.7 (6.8)	−0.02	0.07	−0.09	-	0.41 **	0.26 **	0.02	0.33 **	0.27 **		
5	Job demand (M (SD)) ^‡^	10.1 (1.9)	9.1 (1.8)	−0.22 **	0.15 **	−0.13 *	0.13 *	-	0.47	0.12	0.35 **	0.14 *		
6	Job control (M (SD)) ^‡^	9.1 (2.1)	7.9 (2.3)	−0.15 **	0.11 **	−0.07	−0.01	0.32 **	-	0.40 **	0.36 **	0.03		
7	Social support (M (SD)) ^‡^	7.8 (1.9)	7.6 (1.9)	−0.16 **	−0.02	−0.09	-0.18 **	0.26 **	0.44 **	-	0.31 **	0.08		
8	Job insecurity (M (SD)) ^‡^	4.3 (1.3)	3.4 (1.2)	0.04	0.05	−0.10	0.36 **	0.10	0.25 **	0.18 **	-	0.19 **		
9	Workplace mistreatment (n (%)) ^†^	39 (11.1)	14 (6.0)	−0.09	0.09	−0.11	0.34 **	0.21 **	0.04	0.08	0.27 **	-		
10	Emotional effort (M (SD))	8.6 (1.9)	NA	−0.12 *	0.09	−0.10	0.03	0.34 **	0.27	0.37 **	0.25 **	0.21 **	-	
11	Emotional dissonance (M (SD))	11.9 (3.0)	NA	−0.10	0.08	−0.12	0.29 **	0.30 **	0.14	0.05	0.23 **	0.24 **	0.52 **	-
	Cronbach’s α			-	-	-	0.855	-	0.622	0.719	0.797	0.727	0.808 ^§^	0.730 ^§^

Correlation coefficient for sales workers were presented in the gray color cell. Those for manual workers were in the non-colored. ^†^ *p* < 0.01 from chi-square test, ^‡^ *p* < 0.01 from independent *t*-test, * *p* < 0.05, and ** *p* < 0.05 from Pearson’s correlation test, ^§^ Cronbach alpha values only from sales workers’ data.

**Table 2:** Predictors for depressive symptoms among women workers: hierarchical multiple regression analysis (*N* = 583).

Dependent Variables	Model 1				Model 2				Model 3			
	β	SE	_S_β	sr^2^	β	SE	_S_β	sr^2^	β	SE	_S_β	sr^2^
**Step 1**												
Age	0.03	0.07	0.02	0.000	0.03	0.06	0.02	0.000	0.02	0.06	0.01	0.000
Education	1.06	0.82	0.05	0.003	0.14	0.72	0.01	0.000	0.02	0.72	0.00	0.000
Living alone	−2.58	0.93	−0.12	0.013	−1.34	0.82	−0.06	0.003	−1.34	0.82	−0.06	0.003
Occupation	2.85	0.66	0.18	0.030	1.23	0.62	0.08	0.005	1.25	0.61	0.08	0.005
**Step 2**												
Workplace mistreatment					6.24	1.04	0.23	0.046	6.19	1.04	0.22	0.044
Job demand					0.65	0.17	0.16	0.019	0.71	0.17	0.17	0.021
Job control					0.13	0.16	0.04	0.001	0.10	0.16	0.03	0.001
Social support					−1.01	0.17	−0.24	0.046	−0.095	0.17	−0.23	0.040
Job insecurity					1.82	0.24	0.30	0.073	1.70	0.24	0.28	0.062
**Step 3**												
Occupation×Workplace mistreatment									0.14	0.29	0.02	0.000
Occupation×job demand									−0.39	0.32	−0.05	0.002
Occupation×job control									−0.39	0.34	−0.05	0.002
Occupation×social support									−0.72	0.32	−0.09	0.006
Occupation×job insecurity									0.55	0.32	0.07	0.004
F from ANOVA	8.361 **				24.481 **				16.989 **			
R^2^	0.055				0.278				0.295			
R^2^ change					0.223				0.017			

β: regression coefficient, SE: standard error, _S_β: standardized regression coefficient, sr^2^: squared semipartial correlation coefficients indicate the amount variance uniquely predicted by each predictor. * *p* < 0.05, and ** *p* < 0.01 from multiple regression analyses.

**Table 3:** Predictors for depressive symptoms among sales workers and manual workers: multiple regression analyses stratified by occupation (*N* = 350).

Dependent Variables	Manual Workers (*n* = 233)				Sales Workers (*n* = 350)							
					Model 1				Model 2			
	β	SE	_S_β	sr^2^	β	SE	_S_β	sr^2^	β	SE	_S_β	sr^2^
Age	0.10	0.08	0.08	0.005	−0.03	0.08	−0.02	0.000	−0.03	0.08	−0.02	0.000
Education	−0.05	1.19	0.00	0.000	0.05	0.92	0.00	0.000	0.15	0.90	0.01	0.000
Living alone	−2.69	1.30	−0.14	0.014	−0.74	1.06	−0.04	0.001	−0.54	1.03	−0.03	0.001
Workplace mistreatment	5.38	1.68	0.19	0.034	6.53	1.32	0.24	0.052	6.12	1.30	0.23	0.045
Job demand	0.96	0.25	0.26	0.048	0.53	0.23	0.12	0.011	0.44	0.23	0.10	0.007
Job control	0.32	0.22	0.11	0.007	−0.06	0.22	−0.01	0.000	−0.08	0.22	−0.02	0.000
Social support	−0.45	0.24	−0.12	0.012	−1.26	0.23	−0.29	0.064	−0.98	0.24	−0.22	0.035
Job insecurity	1.16	0.37	0.20	0.032	2.06	0.31	0.33	0.092	1.97	0.31	0.32	0.081
Emotional effort	Not applicable				Not included				−0.75	0.26	−0.17	0.017
Emotional dissonance	Not applicable				Not included				0.66	0.16	0.23	0.036
F from ANOVA	10.311 **				15.838 **				15.166 **			
R^2^	0.269				0.271				0.309			

β: regression coefficient, SE: standard error, _S_β: standardized regression coefficient, sr^2^: squared semipartial correlation coefficients indicate the amount variance uniquely predicted by each predictor. * *p* < 0.05, and ** *p* < 0.01 from multiple regression analyses.
